# Pedestrian Safety Among High School Runners: A Case Series

**DOI:** 10.1177/19417381221123510

**Published:** 2022-09-25

**Authors:** Erin Shore, Randi DeLong, Elaine Powell, Johna Register-Mihalik, Rebecca Stearns, Michael C. Koester, Kristen Kucera

**Affiliations:** † National Center for Catastrophic Sport Injury Research, Department of Exercise and Sport Science, The University of North Carolina at Chapel Hill, Chapel Hill, North Carolina; ‡ Department of Epidemiology, The University of North Carolina at Chapel Hill, Chapel Hill, North Carolina; § Department of Exercise and Sport Science, The University of North Carolina at Chapel Hill, Chapel Hill, North Carolina; ‖ Mathhew Gfeller Sport-Related Traumatic Brain Injury Research Center, Department of Exercise and Sport Science, The University of North Carolina at Chapel Hill, Chapel Hill, North Carolina; ¶ Department of Kinesiology, University of Connecticut, Storrs, Connecticut; # Korey Stringer Institute, University of Connecticut, Storrs, Connecticut; ** Slocum Center for Orthopedics and Sports Medicine, Eugene, Oregon

**Keywords:** youth sports, athletes, pedestrians, motor vehicles, safety

## Abstract

**Background:**

Participation in high school cross-country and track has increased over the last few decades. At the same time, the rate of pedestrian-involved motor vehicle crashes (MVCs) has also increased. In the context of organized sport, pedestrian safety among runners is often not highlighted, despite the risk of catastrophic injury.

**Purpose:**

To describe incidents of pedestrian-involved MVCs involving student athletes captured by the National Center for Catastrophic Sport Injury Research (NCCSIR) at the University of North Carolina at Chapel Hill.

**Study Design:**

Case series.

**Level of Evidence:**

Level 5.

**Methods:**

This study utilized surveillance data from the NCCSIR from 2011 to 2020. It presents descriptive statistics, including frequencies and percentages, detailed summaries, and a Haddon Matrix.

**Results:**

There were 8 incidents involving 11 student athletes, resulting in 9 fatalities. Of these, 5 cases occurred in the afternoon or early evening, 4 occurred in the Fall, and 6 occurred in a rural area. Haddon Matrix analyses of case descriptions indicate schools should implement a runner safety program for all new runners and ensure that runner safety measures are included in emergency action plans.

**Conclusion:**

Runner-related MVCs are relatively rare, but tragic, incidents. Pedestrian safety measures should be incorporated into school-sponsored practices and training runs.

**Clinical Relevance:**

Pedestrian safety should be incorporated into runner safety and injury prevention efforts.

Pedestrian fatalities increased by 45% in the United States from 2009 to 2017, according to the National Highway Traffic Safety Administration.^24^ A pedestrian was killed in a motor vehicle crash (MVC) on average every 88 minutes in 2017.^23^ Despite these alarming statistics, pedestrian safety is often not part of any formal educational curriculum and rarely enters the conversation when it comes to runner safety issues for both competitive and recreational runners. Much of the research on runner safety focuses on biomechanics and musculoskeletal injuries,^7,28^ while pedestrian safety is its own research focus area often not integrated into sports medicine settings. While runner-vehicle collisions remain relatively rare, the outcomes of these events are often catastrophic. Worldwide and in the United States, being a pedestrian is dangerous.^9^

While the incidence of pedestrian fatalities due to an MVC has been increasing over the last decade, participation in high school cross-country and track has also been increasing. According to the National Federation of State High School Associations, participation in US high school cross-country increased by 38% and participation in US track (indoor and outdoor) increased by 23% from the academic year 2002/2003 to 2018/2019.^22^ More high school students participating in cross-country and track means there are more student pedestrians on the road at risk of being involved in an MVC, as many students practice and train by running on or near roads close to their schools. Due to this injury risk, running-related pedestrian safety should be considered in research and prevention efforts.^20^

Research on pedestrian safety over the past decade identified risk factors of pedestrian fatalities due to an MVC, which include failure to obey traffic lights and not using a proper intersection to cross the street.^3,11,14^ Adolescent pedestrians injured in an MVC crossed the street midblock more often than injured adults in the same neighborhood.^8^ Other common risk factors associated with MVC pedestrian fatalities are the type of vehicle involved, with trucks and SUVs being more dangerous,^3,11,23^ and lighting conditions, where darkness was a higher risk.^11,23^ To date, no studies have focused on pedestrian safety of runners in an organized sport context.

Over the last decade, the National Center for Catastrophic Sport Injury Research (NCCSIR) at the University of North Carolina at Chapel Hill captured multiple student athlete fatalities and severe injuries resulting from an MVC during a training session, including 4 incidents involving 7 athletes in 2020. The purpose of this paper is to describe those incidents in detail and highlight commonalities and areas for risk reduction for high school runners.

## Methods

### Study Design

This is a case series study using surveillance data collected by the NCCSIR - a research center based at the University of North Carolina at Chapel Hill. The participants are middle and high school athletes who participated in sponsored athletic programs from 2011 to 2020. This study was approved by the institutional review board of the University of North Carolina at Chapel Hill.

### Data Collection

The NCCSIR has performed surveillance of catastrophic sports injuries among professional, collegiate, high school, and youth sports in the United States since 1982.^21^ Catastrophic sports injuries are defined by the NCCSIR as a fatality; a nonfatal injury with a permanent, severe functional disability; or a serious injury with no permanent functional disability, but still severe, such as a spinal fracture with no paralysis.^15^ The NCCSIR collects data via online news reports, social media, professional associates of the research team, coaches, athletic trainers, athletic directors, and executive officers of state and national athletic organizations.^15^ Data collected by the NCCSIR include details like athlete demographics, injury event, and injury outcome. This case series includes athlete sex, age, grade, sport (cross-country or track and field), type of injury (Other Traumatic > Other Accident > Transportation-Pedestrian), and injury outcome for all instances of high school cross-country and track and field distance runners hit by a vehicle while training from 2011 to 2020. For the purposes of this study, the authors reviewed all relevant media reports and police crash reports, when available, to obtain additional information like time of day and year of the incident, road location, and details about the driver.

### Statistical Analysis

Descriptive statistics included frequencies and percentages. Athlete characteristics included in this case series are athlete sex, age, grade, sport, athlete activity, and injury outcome. Characteristics about the MVC included are time of day, time of year, geographic area, road location of the incident, and driver details.

### Haddon Matrix Analysis

Each incident was summarized and presented to provide context for each incident and injury. Based on these case summaries and the descriptive statistics, a Haddon Matrix was completed to generate ideas for potential interventions. The Haddon Matrix was developed more than 4 decades ago to broaden the thinking about prevention efforts for MVCs. This conceptual model utilizes 3 time frames: pre-event, event, and postevent and focuses on 4 factors: the host (the person at risk), the agent/vehicle, the physical environment, and social environment.^29^ The Haddon Matrix is commonly used in the injury prevention field to brainstorm ideas for interventions to prevent the occurrence of injuries. For the purposes of this study, the focus will be on the host and the social environment and the pre-event phase as areas for potential interventions.

## Results

From 2011 to 2020, the NCCSIR captured 8 MVC incidents involving 11 student athletes. Nine athletes were killed, 1 athlete was permanently disabled, and 1 athlete sustained a serious injury for which a full recovery is expected. Ten of the athletes were running as part of an official school practice and 1 was running as personal training. Of the 11 cases, 4 were from 1 incident where a pickup truck drove onto the sidewalk and struck a group of runners as they were preparing to run.

Of the 8 MVCs, 5 occurred in the afternoon or early evening, 4 occurred in the Fall (September to November), and 6 were in rural areas. The remaining 3 MVCs occurred in an intersection or in the middle of the road, while 4 occurred on the side of the road or sidewalk (Appendix Table A1, available in the online version of this article). Based on data available to the NCCSIR, there were few details about the drivers involved in the MVCs. In one incident, the driver reported looking down at something they dropped and that is when the crash occurred. In another incident, the driver was intoxicated and drove their vehicle onto the sidewalk striking a group of athletes preparing to run. Of the 11 athletes included in this study, 7 were male runners. Of the 8 MVC incidents included, 7 occurred while the athletes were actively running, while the other incident involved athletes that were preparing to run (Appendix Table A1, available online). Their grades in school ranged from 8th to 12th, with 4 being 9th graders and 4 being 12th graders. There was no information available on details about prevention efforts, such as whether or not the athlete was wearing bright colors or reflective materials. Appendix Table A2 (available online) provides detailed summaries of each incident captured by the NCCSIR from 2011 to 2020.

Appendix Table A3 (available online) shows a Haddon Matrix of potential prevention strategies for MVCs involving high school and cross-country track athletes. For the purposes of this study, the intervention strategies pertaining to the person and the social environment and the pre-event stage will be detailed. Interventions aimed at the high school runners themselves before the MVC include implementing a safe running program for new runners and requiring all runners to wear a safety vest. With respect to the school/team environment, potential interventions include working to create a culture of safety among the team and ensuring that emergency action plans (EAPs) and policies include runner safety measures.

## Discussion

While these incidents are relatively rare in the larger context of running-related injuries, it is alarming that the NCCSIR captured 4 incidents of middle or high school cross-country runners involved in an MVC in 2020 alone. This is unfortunately consistent with the increase in pedestrian fatalities over the last decade.^1,24^ While this study was too small to perform test of statistical significance, the fatality rate in this study was quite high (9 of 11 athletes), which is clinically meaningful. There is limited previous research on MVC injuries among runners; these incidents are consistent with previous attempts to describe the incidence of runner involved MVCs. In a study of 65 instances of joggers hit by vehicles over a 1-year period, the researchers found that the age-group of joggers most often hit by a vehicle was 15 to 24 years of age and that such instances occurred in the afternoon, involved runners crossing the street between intersections, and nearly half were killed (30/65).^30^ Although this particular study took place decades ago, the results are similar to those in the current case series, and to other more general pedestrian safety statistics,^3,11,14^ which suggests the underlying safety risks persist and demands further attention despite the safety recommendations made to joggers years ago.^30^ The running community, including school-sponsored cross-country and track, can highlight these prevention measures to assure that runners, specifically young runners, know how to be safe while training.

Currently, there is little, if any, research or guidance specifically for school-sponsored cross-country and track teams with respect to pedestrian safety.^20^ However, many resources and recommendations for general pedestrian safety could be used and adapted to meet the needs of runners. Runners should always use sidewalks, if available, and should run facing traffic. Previous research has shown that nearly all pedestrian fatalities occur at a location other than the sidewalk^23^ and that pedestrians walking facing traffic have a reduced risk of being struck by a motor vehicle.^16^

Runners should cross the street at intersections and cross walks, whenever available. According to the National Highway Traffic Safety Administration, nearly three-fourths of all pedestrian fatalities in 2017 occurred in a location other than an intersection.^23^ Indeed, in this case series, 5 of the 8 crashes occurred at a location other than an intersection.

Runners should avoid running in the dark.^30^ In 2017, 75% of the pedestrian fatalities occurred in the dark.^23^ Previous research has confirmed that the risk of a pedestrian-involved MVC was greater in the dark compared with in daylight.^12,27^ In this study, many incidents occurred in the early morning, late afternoon, or early evening, where light and visibility could have been a factor. In addition to avoiding running in the dark, runners should be encouraged to wear bright, reflective clothing.^30^ Specifically, the students should wear reflective clothing on their limbs, including wrists, ankles, and other major joints compared with around the torso, as this has been shown to help drivers distinguish pedestrians in low-light conditions.^17^ As presented in the Haddon Matrix, schools could mandate that all student athletes wear bright, neon clothing while participating in school-sanctioned practice runs where the students would be running in public. If the students do not have clothing that meets this requirement, the school could provide a neon, reflective vest or reflective bands to wear on their limbs, to increase the runners’ visibility while they are out running.

The adapted resources and recommendations for runner pedestrian safety presented here could be packaged as a training that would be mandated for all school-sponsored running, as suggested in the Haddon Matrix. The training could also include suggestions like warming up and cooling down at locations other than by the road or on the sidewalk to avoid incidents like one included in this study, where students were struck on a sidewalk while preparing to run. Such training could include best practices for running in public, such as running against traffic, knowing when and where it is appropriate to cross the road, not wearing headphones, etc, and could be implemented at the beginning of each cross-country and track season.

The aforementioned recommendations could be promoted through school policy changes and an educational campaign advocating for a safer running environment. In other high school sports, policy changes have been successful in reducing risk. For example, after the state-mandated implementation of the National Athletic Trainers’ Association Inter-Association Task Force’s preseason heat acclimatization guidelines, the rates of exertional heat-related illness among high school athletes participating in preseason sports activities were lower than before the guidelines went into effect.^13^ In addition, pedestrian safety educational programs have proven successful among school-aged children.^6,18,19^ For example, a school-based education program in Canada demonstrated improved knowledge and self-reported pedestrian behaviors after a single session education program,^19^ and an interactive education program in Los Angeles was associated with improved knowledge, safe behaviors, and fewer injuries.^18^ Policy changes with respect to rules and expectations around runner safety during practice would fall into the social environment/pre-event phase of the presented Haddon Matrix. These changes would also contribute to the culture of safety among teams. A positive safety climate is associated with better safety compliance among workers,^4^ so a positive safety climate among a team could help assure that all runners are following the road-safety rules. It should not only be expected but encouraged that all student athletes will follow safety guidelines. Coaches, athletic trainers, and even team captains should demonstrate safe behavior and encourage others to behave in the same manner.

In addition, as noted in the Haddon Matrix, runner safety measures should be included in the school’s EAP and there should be an annual review of the EAP, including drills, as suggested in the Haddon Matrix. Having an EAP on file is the recommended best practice for high school athletics to prevent sudden death.^2^ According to a survey of athletic trainers, a vast majority (89%) reported having an EAP on file, with nearly 80% reporting they update the EAP annually.^25^ Adding runner safety measures to the EAP is a simple step that could help bring awareness to runner safety issues and prepare coaches and athletic trainers on how to respond in an emergency situation involving a runner-related MVC.

These kinds of catastrophic events impact more than just the athlete involved. The athletes who lose a teammate or witness their teammate being struck by a motor vehicle might suffer psychological distress. A qualitative study of college athletes who lost a teammate showed they went through a range of emotions including denial, shock, grief, and anger, among others.^26^ Another qualitative study among gymnasts who witnessed injuries of their teammates described experiencing somatic symptoms like nausea, fatigue, and panic attacks and intrusive thoughts about their injury risk.^5^ Similarly, a study of adolescent MVC survivors found an association between an MVC and depression and alcohol abuse.^31^ Another study among college students who were experiencing bereavement after a fatal MVC of a loved one found that 30% of the individuals suffered post-traumatic stress disorder.^10^ Preventing pedestrian-involved MVCs among school-aged runners would also prevent young athletes from experiencing the negative psychological effects of witnessing such an incident or losing a teammate.

Strengths of this case series include the scope and breadth of the data collected by the NCCSIR. The data collected by the NCCSIR are nationally representative, so the cases presented can be instructive for the whole country. Another strength is the amount of data collected for each injury. Because this study used media reports, particularly local media reports, and in some cases crash reports, to gather information, many details about each incident were able to be learned, such as the time of day and specific location of each incident. However, there are limitations of this study. The data presented are likely an undercount of all cross-country and track runners who were involved in an MVC incident. The NCCSIR captures only catastrophic injuries, so the number of students who sustained minor injuries or serious but nonlife-threatening injuries from an incident like this is not known. Another limitation is that because the NCCSIR relies so heavily on media reports, if the media did not cover an incident where a student runner was involved in an MVC, it is likely that case is not recorded. However, national statistics do not distinguish between runners and other pedestrians, which has not changed for decades,^30^ so these are still the best data available.

## Conclusion

Although runner-vehicle incidents are relatively rare in terms of runner injuries, the outcomes of these incidents are severe and often fatal, as shown in this case series. The dangers to young, inexperienced runners are real and need to be highlighted before students are sent out to run on the road. Pedestrian safety should be included in runner safety in general, but specifically for school-sponsored practice and training runs.

## Supplemental Material

sj-docx-1-sph-10.1177_19417381221123510 – Supplemental material for Pedestrian Safety Among High School Runners: A Case Series Click here for additional data file. Supplemental material, sj-docx-1-sph-10.1177_19417381221123510 for Pedestrian Safety Among High School Runners: A Case Series by Erin Shore, Randi DeLong, Elaine Powell, Johna Register-Mihalik, Rebecca Stearns, Michael C. Koester and Kristen Kucera in Sports Health: A Multidisciplinary Approach
