# Supplementary material for: Pedestrian Safety Among High School Runners: A Case Series
Source: Sports Health. 2022 Sep 25;15(5):633–7. doi: 10.1177/19417381221123510 (PMC10467471; doi:10.1177/19417381221123510)
Supplement: sj-docx-1-sph-10.1177_19417381221123510 – Supplemental material for Pedestrian Safety Among High School Runners: A Case Series [file sj-docx-1-sph-10.1177_19417381221123510.docx]

APPENDIX

| Table A1. Characteristics of MVCs and high school athletes involved in an MVC while running from 2011-2020 captured by the NCCSIR. | | |
| --- | --- | --- |
| MVC Incidents (N=8) | | |
| MVC Characteristic | | N (%) |
| Time of Day | |  |
| Morning | | 3 (37.5%) |
| Afternoon | | 5 (62.5%) |
| Time of Year | |  |
| Summer (June – August) | | 2 (25.0%) |
| Fall (September – November) | | 4 (50.0%) |
| Winter (December – February) | | 1 (12.5%) |
| Spring (March – May) | | 1 (12.5%) |
| Multiple pedestrian injuries/deaths | | 1 (12.5%) |
| Geographical Area | |  |
| Rural | | 6 (75.0%) |
| Suburban | | 2 (25.0%) |
| Road Location of Incident | |  |
| Side of road | | 4 (50.0%) |
| Intersection | | 3 (37.5%) |
| Middle of road | | 1 (12.5%) |
| Driver details | |  |
| Driver impaired | | 1 (12.5%) |
| Driver distracted | | 1 (12.5%) |
| Unknown | | 6 (75.0%) |
| Athlete Characteristics (N=11) | | |
| Athlete Characteristic | | N (%) |
| Sex | |  |
| Female | | 4 (36.4%) |
| Male | | 7 (63.6%) |
| Athlete Age | |  |
| 13 | | 2 (18.2%) |
| 14 | | 2 (18.2%) |
| 16 | | 2 (18.2%) |
| 17 | | 2 (18.2%) |
| 18 | | 1 (9.1%) |
| Unknown | | 2 (18.2%) |
| Athlete Grade | |  |
| 8^th^ grade | | 1 (9.1%) |
| 9^th^ grade | | 4 (36.4%) |
| 10^th^ grade | | 2 (18.2%) |
| 12^th^ grade | | 4 (36.4%) |
| Injury Sport | |  |
| Cross-country | | 6 (54.5%) |
| Track | | 5 (45.5%) |
| Action | |  |
| Running middle/long distance | | 7 (63.6%) |
| Getting ready/preparing for run | | 4 (36.4%) |
| Injury Outcome | |  |
| Fatality | | 9 (81.8%) |
| Trauma-related non-fatality with permanent  disability | | 1 (9.1%) |
| Trauma-related non-fatality with disability  unknown/uncertain | | 1 (9.1%) |
| Table A2. Detailed case summaries of incidents of high school athletes being struck by a motor vehicle during a training run from 2011 to 2020 captured by the NCCSIR. | | |
| Incident 1: | An incoming high school female freshman cross country runner was struck and killed by a vehicle during an early morning practice. The athlete was running with her cross-country team in a rural area. The crash happened at approximately 7:15 am and occurred when the athlete was attempting to cross the highway. The driver was 27 years old and no charges were filed against him. There was no information about the weather, the clothing of the athlete, nor what the driver was doing at the time of the crash. | |
| Incident 2: | A high school female freshman was struck and killed while running in a rural area during cross-country practice after school. The team was instructed to run for thirty minutes and the athlete and another student ran down a state highway, where the athlete was struck by the car while trying to cross the road. There was a pronounced dip in the road, which may have obscured the athletes from the driver’s view. There were no charges filed against the 51-year-old driver. There was no information on which side of the road the athlete was running, where she tried to cross the road, the athlete’s clothing, nor the actions of the driver at the time of the incident. | |
| Incident 3: | A high school male freshman was running with his cross-country team before school when he was struck by a vehicle while crossing the street at an intersection. The team was running with their coach when they came to the intersection and the ‘do not cross’ symbol was illuminated. The coach allegedly said “Let’s go” and the team crossed the intersection. The athlete was at the back of the pack and was hit by a car. He spent ten days in a coma and suffered a traumatic brain injury that left him permanently disabled. His family sued the coach and was awarded a settlement from the school district. There was no information regarding the athlete’s clothing. | |
| Incident 4: | A middle school male eighth grade student was running on a trail in a rural area with his cross-country team after school when he was struck and killed by a car while trying to cross a highway. The driver of the car reportedly looked down momentarily and looked up to see the athlete in front of his car when he slammed on the brakes and steered left into the shoulder to try and avoid the athlete. The driver got out of the car, called 911, and tried to help until EMS arrived on the scene. The athlete was air lifted to a hospital where he died the next day. The trail that the athlete was running on did not have any stop signs for pedestrians at the intersection with the highway and was situated just outside of town near where the speed limit increased from 25mph to 55mph. No charges were made against the driver. There was no information regarding the athlete’s clothing. | |
| Incident 5: | A high school male sophomore was struck by a car while running on the sidewalk near his suburban high school during an after-school track practice. The athlete was reportedly doing all the correct things – running on the sidewalk and following all the road rules. The athlete was struck when a truck drove onto the sidewalk and collided with him. The driver stayed on the scene and cooperated with law enforcement. There was no evidence of alcohol or drug use in this case. At this time, there have been no charges filed against the driver. There was no information regarding the athlete’s clothing. | |
| Incident 6: | A high school male freshman cross-country athlete was struck and killed after a 2-car collision in a rural area. The athlete was running on a local highway in the morning with other athletes at the time of the crash. A car tried to turn left and hit another car, which then spun out of control and struck the athlete. The school resource officer ran to the scene and alerted EMS. The athlete was air lifted to a hospital where he died from his injuries. At this time there have been no charges filed against either of the drivers involved. There was no information regarding where on the road the athlete was or if they were wearing any bright, reflective clothing. | |
| Incident 7: | A group of high school track and field athletes were preparing for a warm-up run on the sidewalk for an after-school practice when a pickup truck drove up onto the sidewalk and struck them. There were seven athletes involved in the accident. The driver was driving close to 80mph in a 25mph speed zone and he kept driving after colliding with the athletes. Three of the seven athletes sustained injuries that were not considered catastrophic, so they are not included in this study. They were treated and released from the hospital. Details of each of the catastrophically injured athletes are provided below.   - A high school female senior died at the scene of the incident. - A high school female sophomore was transferred to the hospital via EMS where she later died of her injuries. - A high school male senior was transferred to the hospital via EMS and was in critical condition for two weeks before he died of his injuries. - A high school male senior was transferred to the hospital via EMS where he was treated for bruised lungs and other injuries including broken bones and damaged ligaments. | |
| Incident 8: | A high school male senior was running along a highway just after 5:00 pm. The athlete crossed the highway and jumped over the median barrier in the highway when he was struck by oncoming traffic. The athlete died at the scene from blunt force injuries. No information has been released about the driver, nor were there details about whether the athlete was wearing bright, reflective clothing or not. | |

| Table A3. Haddon Matrix for the prevention of MVCs involving high school cross-country and track athletes | | | | |
| --- | --- | --- | --- | --- |
|  | Person (victim)  *High school runner* | Agent (energy)  Vector (animate)  Vehicle (inanimate)  *Motor vehicle* | Physical Environments  *Road/trail student is*  *running on* | Social Environments  *School/team environment* |
| Pre-event  *Before MVC* | Implement safe running  training program for new  runners  Required to wear a  safety vest or reflective clothing/markers | Install sensors in all cars that detect pedestrians  Reduce distracted driving | Improve/maintain the quality of sidewalks  Create trails for students that don’t intersect roads | Create a culture of following safety guidelines  Ensure running safety measures are included in emergency action plans and policies |
| Event  *MVC* | Fewer distractions  Wearable device that could sense the crash and alert EMS | Restrict motor vehicles to smaller sizes  Reduced speed | Improve/maintain road conditions where students will be running  Resurface roads/sidewalks with a softer surface | All students should run with a cell phone  Training in remaining calm in emergencies |
| Post-event  *Immediately*  *Following MVC* | Call EMS, if able  Move to safety, if able | Install emergency services contact in every motor vehicle  Install a first aid kit and AED in every car | Install emergency call buttons/stations along running routes  Install first aid kits/AEDs along roads | Have an emergency action plan and make sure students know the plan  CPR/First Aid/AED education and training |
